# Diagnostic dose determination and efficacy of chlorfenapyr and clothianidin insecticides against *Anopheles* malaria vector populations of western Kenya

**DOI:** 10.1186/s12936-019-2858-z

**Published:** 2019-07-17

**Authors:** Silas Agumba, John E. Gimnig, Lilian Ogonda, Maurice Ombok, Jackline Kosgei, Stephen Munga, Benard Guyah, Seline Omondi, Eric Ochomo

**Affiliations:** 1 Maseno University, Private Bag, Maseno, Kenya; 2 Centre for Global Health Research, Kenya Medical Research Institute, PO Box 1578-40100, Kisumu, Kenya; 3 Division of Parasitic Diseases and Malaria, Center for Global Health, Centers for Disease Control and Prevention, Atlanta, GA 30333 USA

**Keywords:** *Anopheles*, *Anopheles gambiae* sensu stricto, *Anopheles arabiensis*, *Anopheles funestus*, Chlorfenapyr, Clothianidin, Insecticide resistance

## Abstract

**Background:**

Malaria vector control is dependent on chemical insecticides applied to walls by indoor residual spraying or on long-lasting insecticidal nets. The emergence and spread of insecticide resistance in major malaria vectors may compromise malaria control and elimination efforts. The aim of this study was to estimate a diagnostic dose for chlorfenapyr (class: pyrrole) and clothianidin (class: neonicotinoid) and assess the baseline susceptibility of three major *Anopheles* malaria vectors of western Kenya to these two insecticides.

**Methods:**

The Centers for Disease Control and Prevention (CDC) bottle assay was used to determine the diagnostic doses of chlorfenapyr and clothianidin insecticides against the susceptible Kisumu strain of *Anopheles gambiae*. Probit analysis was used to determine the lethal doses at which 50% (LD50) and 99% (LD99) of the susceptible mosquitoes would be killed 24, 48 and 72 h following exposure for 1 h. Insecticidal efficacy of chlorfenapyr, clothianidin and the pyrethroid deltamethrin was then evaluated against field collected female *Anopheles* mosquitoes sampled from Nyando, Bumula and Ndhiwa sub-Counties in western Kenya. Members of *Anopheles funestus* and *An. gambiae* complexes were identified using polymerase chain reaction (PCR).

**Results:**

The determined diagnostic doses of chlorfenapyr and clothianidin insecticides were 50 µg/bottle and 150 µg/bottle, respectively, for *An. gambiae*, Kisumu strain. When exposed to the diagnostic dose of each insecticide, *Anopheles* malaria vector populations in western Kenya were susceptible to both insecticides with 100% mortality observed after 72 h. Mortality of mosquitoes exposed to deltamethrin increased over time but did not reach 100%. Mortality of *Anopheles arabiensis* from Nyando exposed to deltamethrin was 83% at 24 h, 88% at 48 h and 94.5% at 72 h while *An. funestus* from Ndhiwa was 89% at 24 h, 91.5% at 48 h and 94.5% at 72 h.

**Conclusion:**

Mosquitoes of western Kenya, despite being resistant to pyrethroids, are susceptible to chlorfenapyr and clothianidin. Field evaluations of the formulated product are needed.

**Electronic supplementary material:**

The online version of this article (10.1186/s12936-019-2858-z) contains supplementary material, which is available to authorized users.

## Background

Considerable progress in reducing the global burden of malaria has been achieved since 2000. In Africa, prevalence estimated from national surveys declined by half from 2000 to 2015 while clinical cases declined by 40% [1]. More recently, gains in reducing malaria have slowed and in some countries, malaria burden has increased [2]. Sub-Saharan Africa shoulders the bulk of the malaria burden with 88% of malaria cases and 93% of malaria deaths occurring in this region [3]. There are likely multiple reasons for the slowed progress in reducing malaria, but one likely reason is the emergence and spread of insecticide resistance in the major malaria vectors, a development that may compromise chemical based malaria control interventions and thereby threaten malaria control and elimination efforts.

There are six classes of insecticides recommended for malaria vector control: pyrethroids, organochlorines, carbamates, organophosphates, pyrrole and neonicotinoids [4]. Pyrethroids are widely used for malaria vector control but resistance has developed in the major malaria vectors of Africa [5] and is now present in nearly every country in sub-Saharan Africa [6] (http://www.irmapper.com/). Due to the threat posed by insecticide resistance, there has been an urgent call for alternative insecticides to supplement malaria vector control [7, 8]. As new compounds are developed, it is essential to establish diagnostic concentrations to determine baseline susceptibility of malaria vectors and to enable surveillance of insecticide resistance once the insecticides are in use to guide National Malaria Control Programmes in the deployment and replacement of the insecticide-based vector control tools.

Chlorfenapyr is a slow acting toxin that acts by disrupting respiratory pathways and proton gradients through the uncoupling of oxidative phosphorylation in mitochondria [9]. It has a unique mode of action compared to insecticides currently used for public health; so far, shown there has been no evidence of cross resistance with insect neurotoxins that have widely been used for malaria vector control. Chlorfenapyr is an active ingredient (along with alpha-cypermethrin) on the Interceptor^®^ G2 (BASF, Ludwigshafen Germany), a long-lasting insecticidal net that was recently recommended by the WHO Pesticide Evaluation Scheme [10]. In experimental hut trials, the Interceptor G2 caused higher mortality of wild mosquitoes compared to the WHOPES recommended standard Interceptor^®^ net [11].

Clothianidin is a neonicotinoid insecticide which is chemically similar to nicotine. It acts on the central nervous system of insects as an agonist of acetylcholine and stimulates nicotine acetylcholine receptors (nAChR) [12] activating post-synaptic acetylcholine receptors but does not inhibit acetylene cholinesterase (ACh). High levels overstimulate and block the receptors, [13] causing paralysis and death [12]. Clothianidin is the active ingredient in SumiShield (developed by Sumitomo Chemical Company, Japan) and Fludora^®^ Fusion (Bayer CropScience, Monheim, Germany) along with deltamethrin, an IRS formulation which was recently added to the WHO pre-qualification list of recommended insecticides [14]. This study estimated the diagnostic doses of chlorfenapyr and clothianidin insecticides in bottle bioassays using laboratory-reared *Anopheles gambiae*, Kisumu strain, and used the diagnostic dose identified to evaluate the susceptibility of wild *Anopheles* malaria vectors of western Kenya to chlorfenapyr, clothianidin and the pyrethroid deltamethrin.

## Methods

### Study sites

The study was conducted in three sub-Counties in western Kenya: Ndhiwa in Homa Bay County, Nyando in Kisumu County and Bumula in Bungoma County. The three sites experience perennial transmission of malaria with seasonal peaks between April to July and November to December coincident with the long and short rainy seasons respectively [15]. Malaria prevalence among children 6 months to 14 years as estimated in the most recent Malaria Indicator Survey was 38% for the region despite high coverage with long-lasting insecticidal nets (LLINs) [16]. Most people live in traditional houses with mud walls plus thatched or corrugated iron roofs and practice agriculture as the major economic activity. The main crops grown in Ndhiwa sub-County are sugarcane and maize [17]. The primary malaria vector observed in this site is *Anopheles funestus*. Residents of Bumula sub-County grow cash crops such as sugar cane and tobacco and does horticulture farming. *Anopheles gambiae* sensu stricto (s.s.) is the primary vector in this area [18]. Nyando sub-county is a rice growing area providing larval habitats for *Anopheles arabiensis* throughout the year [19]. Pyrethroid resistance is widespread in western Kenya and has been observed in all three of the primary malaria vector species [20, 21].

### Mosquito sampling and processing

*Anopheles gambiae*, Kisumu strain (pyrethroid susceptible) was reared at the Kenya Medical Research Institute’s Center for Global Health Research (KEMRI-CGHR) insectary in a standard environment. Rearing methodology was based on Ochomo et al. [19] protocol. Wild adult *Anopheles* mosquitoes were sampled indoors using mouth aspirators while larvae were collected from small pools of water using standard dippers. Samples were transported to the insectary at KEMRI-CGHR and identified to species level using morphological keys [22]. Adult mosquitoes collected by indoor aspiration were held for 48 h post collection and then exposed to insecticides. Larvae were reared to adults and exposed when they were 3–5 days old. Members of the *An. gambiae* [23] and *An. funestus* complex [24] were identified by the polymerase chain reaction using primers specific for species known to occur in western Kenya. Mosquitoes from all sites were exposed to chlorfenapyr, clothianidin and deltamethrin insecticides for 1 h. The diagnostic dose for deltamethrin was 12.5 µg/ml, as recommended by WHO for monitoring resistance in the laboratory condition [25].

### Insecticides

Technical-grade active ingredients of chlorfenapyr and clothianidin (Chem Service, Inc., West Chester, PA, USA) were used. Stock solutions were prepared for each insecticide by diluting the active ingredient in absolute ethanol, and storing in glass bottles, wrapped in aluminum foil, and at 4 °C while not being used. Working solutions were prepared from the stock solution. Field collected mosquitoes from two sites (Nyando and Ndhiwa) were exposed to a pyrethroid (deltamethrin) insecticide for comparison.

### Determination of the diagnostic doses of chlorfenapyr and clothianidin

Mosquitoes were tested using the CDC bottle assay following Brodgon and Chan [26]. Given the slow rate of activity of these two insecticides [27, 28], mortality was recorded at 24-h intervals up to 72 h. Ranges of insecticide concentrations were tested for chlorfenapyr (0, 10, 20, 30, 40, 50, 60, 70, 80, 90, and 100 µg/ml) and clothianidin (0, 50, 100, 150, 200 and 250 µg/ml). Approximately 14 h after coating bottles with insecticide, 25 female adult *An. gambiae*, Kisumu (pyrethroid susceptible) (3–5 days old) were aspirated from the main colony and gently blown into each bottle. Mosquitoes were aspirated into the control bottle first, followed by the four insecticide-coated bottles. Once aspirated into each of the five bottles, the timer was started and time zero was recorded. The number of live and knocked down mosquitoes were recorded every 10 min for the 60 min exposure period. After 60 min, the mosquitoes were gently aspirated from the bottle into clean paper cups, and provided with 10% sugar solution soaked in cotton wool during recovery period. All bottles were held vertically for the duration of the experiment. Dose response information of all tested concentration for both chlorfenapyr and clothianidin insecticides is found in Additional file 1.

### Statistical analysis

Data from CDC bottle bioassays were subjected to log probit regression analysis [29] using SPSS statistics 20.0 v and LC50 and LC99 were calculated with 95% confidence intervals. Efficacy of these compounds against wild *Anopheles* was calculated as percentage mortality, following World Health Organization (WHO) guidelines on insecticides susceptibility: mortality ≥ 98% indicates susceptibility, mortality less than 90% indicates the existence of resistance while if the observed mortality is between 90 and 97%, the presence of resistant genes in the vector population must be confirmed [25].

## Results

### Diagnostic dosage of chlorfenapyr and clothianidin insecticides using a susceptible strain

**Fig. 1:**
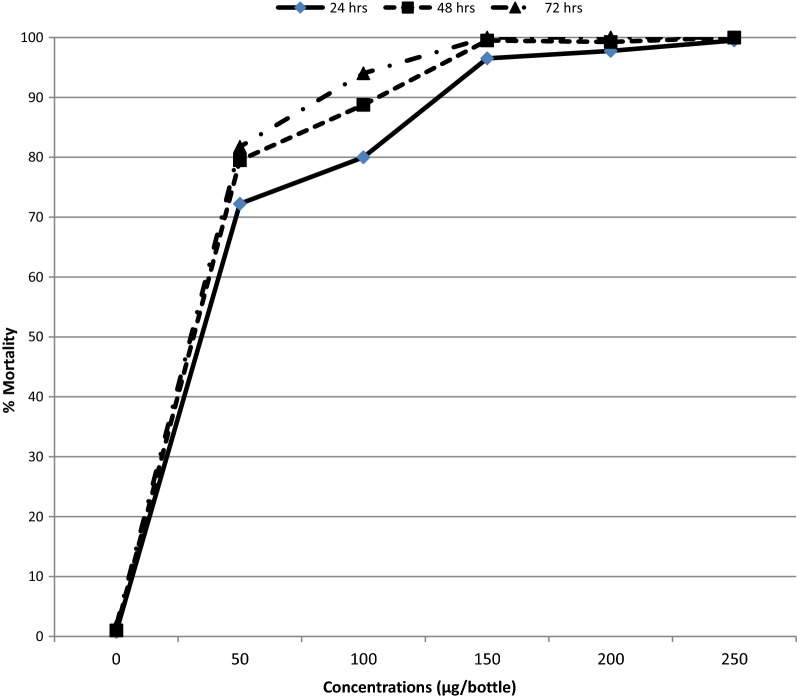
Mortality % of *An. gambiae* s.s (Kisumu strain) to varying concentrations of clothianidin insecticides

**Fig. 2:**
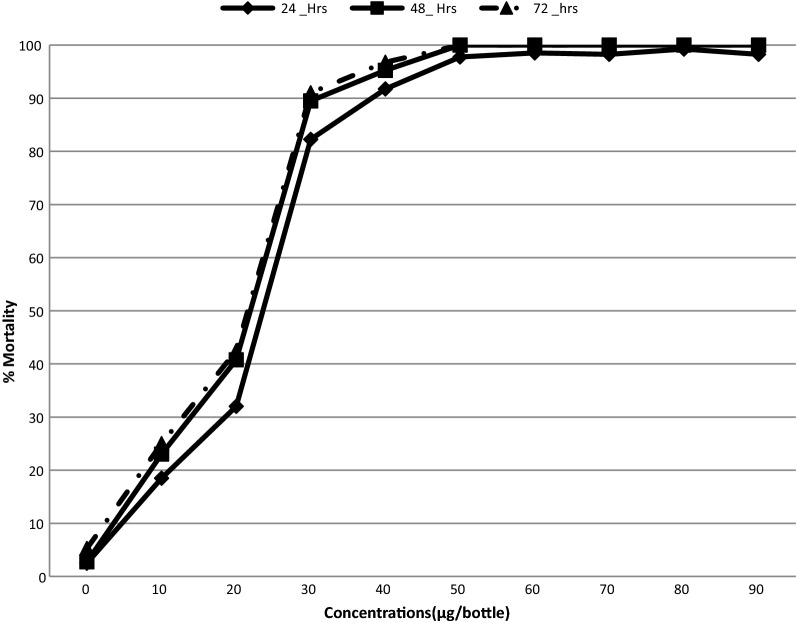
Mortality percentage of *An. gambiae* s.s (Kisumu strain) to varying concentrations of chlorfenapyr

Mortality of *An. gambiae* Kisumu strain exposed to clothianidin and chlorfenapyr is presented in Figs. 1 and 2 respectively. The LC_50_ and LC_99_ for both insecticides estimated by probit analysis are presented in Table 1. The LC50 for chlorfenapyr at 72 h was 55.4 µg/bottle (p-value < 0.001) while the LC50 for clothianidin was 143.5 µg/bottle (p-value = 0.04). For simplicity, the diagnostic doses selected for testing wild mosquitoes were 50 µg/bottle for chlorfenapyr and 150 µg/bottle for clothianidin. Chlorfenapyr had no knockdown effect upon the susceptible *An. gambiae* Kisumu strain after exposure while clothianidin had a mean knockdown of 11.2% at 60 min.

**Table 1:** Probit analysis results of *An. gambiae* s.s. mortality 72 h after exposure to chlorfenapyr and clothianidin

	Chlorfenapyr	Clothianidin
Number exposed	400	400
LD_50%_ (95% CI^a^)	16.8 (13.6–19.9)	27.576 (5.8–41.2)
LD_99%_ (95% CI^a^)	55.4 (42.8–85. 7)	143.5 (100.2–503.9)
Diagnostic dose	50 µg/ml	150 µg/ml
Chi square (χ^2^)	117.5	13.4
p-value	0.001	0.040

^a^Upper and lower limits with 95% confidence intervals

χ^2^ (Chi square)

### Efficacy of chlorfenapyr, clothianidin and deltamethrin against malaria vector populations of western Kenya

**Fig. 3:**
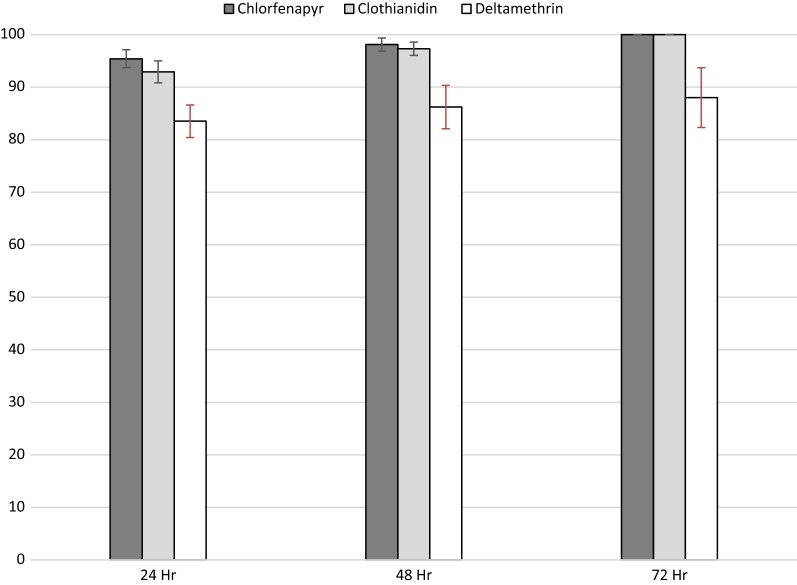
Susceptibility of wild *Anopheles* mosquitoes to chlorfenapyr, clothianidin and deltamethrin insecticides. Mean mortality presented is for 1 h exposure with 95% confidence interval

A total of 2379 mosquitoes were tested for susceptibility to chlorfenapyr, clothianidin and deltamethrin by PCR as shown in Fig. 3, all mosquitoes tested from Nyando sub-county were *An. arabiensis* (1133/1133), 96.5% (386/400) of mosquitoes tested from Bumula sub-County were *An. gambiae* s.s. and 3.5% (14/400) were *An. arabiensis* while 97.3% (827/850) of mosquitoes tested from Ndhiwa sub-county were *An. funestus* and 2.7% (23/400) were *An. arabiensis*. *Anopheles* populations of western Kenya showed 100% mortality in CDC-Bottle bioassays at 72 h post-exposure to both chlorfenapyr and clothianidin with resistance observed to deltamethrin insecticide in two study sites, even at 72 h post-exposure. Mortality 72 h after deltamethrin exposure was 90.0% among *An. arabiensis* in Nyando sub-county and 94.5% among *An. funestus* in Ndhiwa sub-County. Chlorfenapyr had no knockdown effect on wild mosquitoes with 0% knockdown at 60 min in all three populations tested. The mean knockdown rates at 60 min of wild *An. arabiensis*, *An. funestus* and *An. gambiae* s.s. exposed to clothianidin were 12.5%, 4.3% and 0.5% respectively. Knockdown at 60 min after exposure to deltamethrin was 90.5% among *An. arabiensis* from Nyando and 86% among *An. funestus* from Ndhiwa. Table 2 shows results for different holding times by sub-County, which reflect different species compositions.

**Table 2:** Percentage mortality of wild mosquito populations in response to chlorfenapyr, clothianidin and deltamethrin diagnostic doses

Site/population	Insecticide	Sample size (N)	% Knockdown at 60 min	% Mortality		
				24 h	48 h	72 h
Nyando *An. arabiensis*	Chlorfenapyr	407	0	94.6	96.1	100.0
	Clothianidin	415	12.5	92.8	99.3	100.0
	Deltamethrin	200	90.5	83.0	88.0	90.0
Ndhiwa *An. funestus*	Chlorfenapyr	343	0	98.0	99.4	100
	Clothianidin	303	4.3	89.0	97.0	100
	Deltamethrin	200	86.0	89.1	91.5	94.5
Bumula *An. gambiae* s.s.	Chlorfenapyr	200	0	92.7	98.0	100
	Clothianidin	200	0.5	98.1	99.5	100
	Deltamethrin	ND	ND	ND	ND	ND

## Discussion

As new active ingredients are developed, it is essential to establish diagnostic concentrations to determine baseline susceptibility of malaria vectors and to enable monitoring of insecticide resistance once the insecticides are in use to guide National Malaria Control Programmes in the deployment, rotation and replacement of the insecticide based vector control tools. This study determined the diagnostic doses of chlorfenapyr (a pyrrole) and clothianidin (a neonicotinoid) insecticides in CDC bottle bioassays using laboratory-reared *An. gambiae*, Kisumu strain. This study used the diagnostic dose to evaluate the susceptibility of three wild *Anopheles* malaria vectors of western Kenya. The diagnostic doses for chlorfenapyr and clothianidin were defined as the lowest concentration that achieves 100% mortality within 72 h after a 60 min exposure. Based on this criterion, the diagnostic doses were determined to be 50 µg/bottle for chlorfenapyr and 150 µg/ml for clothianidin. These diagnostic doses are important in providing baseline data for monitoring insecticides resistance when deployed for malaria vector control in western Kenya. Three field populations of primarily *An. gambiae*, *An. arabiensis* and *An. funestus*, respectively, were subsequently exposed to these diagnostic doses resulting in 100% mortality at 72 h for both insecticides in all three species tested, confirming baseline susceptibility.

The knockdown rate for both the laboratory strain of *An. gambiae*, Kisumu strain and field collected mosquitoes at 60 min after exposure was low for chlorfenapyr and clothianidin, despite 100% mortality at 72 h. In comparison, pyrethroid insecticides are known for their strong knockdown effects. Even among field populations of *An. arabiensis* and *An. funestus* with some resistance to pyrethroids, exposure to deltamethrin resulted in 90.5% and 86% knockdown, respectively, at 60 min. While the rapid knockdown and killing effect is often considered an important component of the efficacy of pyrethroids, modelling studies have suggested that this mode of action is more likely to select for insecticide resistance [30]. In contrast, slow acting insecticides such as clothianidin and chlorfenapyr could impose less selection pressure for resistance as mosquitoes may survive long enough to lay eggs. Despite the slow acting effect of chlorfenapyr and clothianidin, these insecticides may be able to reduce malaria transmission as mortality was 100% at 72 h in all populations tested and the external incubation period of *Plasmodium falciparum* is a minimum of 8 days [31]. For deltamethrin, mortality did not substantially increase over 72 h suggesting that mosquitoes that survive initial exposure to pyrethroids may be able to sustain transmission of malaria [32].

Resistance to pyrethroid insecticides in western Kenya was first reported in *An. gambiae* s.s. following the implementation of small-scale trials of permethrin treated nets [33]. Initial studies indicated that elevated oxidase and esterase enzymes were associated with increased permethrin tolerance [34]. Subsequently, a target site mutation (*kdr* L1014S) was described in western Kenya and was associated with pyrethroid resistance [8]. Following the scale-up of insecticide treated nets in Kenya, the frequency of the L1014S mutation increased from < 5% to near fixation in *An. gambiae* s.s. [15]. Resistance to pyrethroids was subsequently reported in *An. arabiensis* and *An. funestus* [19, 35]. The resistance mechanisms in *An. arabiensis* and *An. funestus* are not well described, but are likely metabolic detoxification enzymes as the *kdr* mutations in *An. arabiensis* are rare in western Kenya [36] and have never been reported from *An. funestus*. The high levels of susceptibility of field populations to chlorfenapyr and clothianidin indicate a lack of cross-resistance with pyrethroid insecticides. The target sites of both insecticides are different from that of pyrethroids and the lack of cross-resistance indicates that enzymes involved in the metabolic detoxification of pyrethroids do not affect either chlorfenapyr or clothianidin. Furthermore, chlorfenapyr is considered a pro-insecticide that is activated by oxidase enzymes suggesting a potential for negative cross-resistance [37]. Resistance to chlorfenapyr and clothianidin have not yet been described in mosquitoes; but additional information on the molecular mechanisms of insecticide resistance may help to predict the potential for cross-resistance and to guide National Malaria Control Programmes in the selection of insecticides for ITNs and IRS.

The Global Plan for Insecticide Resistance Management (GPIRM) has four main strategies (rotation of insecticides, combination of interventions, mosaic spraying and mixtures) for vector control. Until 2017, only four classes of insecticides were recommended for use in public health—pyrethroids, organochlorines, organophosphates and carbamates; resistance has been reported for each class [14]. There are now two additional classes of insecticides available for use in public health and there are already products formulated with these insecticides. The Interceptor G2 (BASF, Ludwigshafen Germany) is an LLIN treated with alpha cypermethrin plus chlorfenapyr. Experimental hut studies indicate significantly higher mosquito mortality due to the Interceptor G2 compared to pyrethroid only nets, even after washing [11]. SumiShield (Sumitomo Chemical, Japan) and Fludora^®^ Fusion (Bayer Environmental Science, Germany) are IRS products with clothianidin as the active ingredient. SumiShield was demonstrated to last up to 6 months in various surfaces in experimental huts [38]. With the availability of an LLIN treated with a pyrethroid plus chlorfenapyr and two IRS products with clothianidin along with LLINs treated with a synergist (piperonyl butoxide), management of insecticide resistance among wild *Anopheles* populations is more feasible. However, despite general recommendations from the GPIRM, specific guidance is lacking on how to optimally deploy these tools.

The CDC bottle bioassay used for this study offers important advantages. The bottle bioassay allows for substantial flexibility, particularly for assessing resistance to new insecticides, which is useful since diagnostic doses and papers for the WHO tub assay are not yet available. Nevertheless, the approaches used in this study also have limitations. Clothianidin has poor solubility in most solvents used in bottle assays. Clothianidin has very low solubility in acetone and preliminary studies using this solvent have given widely inconsistent results, presumably due to an uneven coating of the bottle. For this reason, the study choose to use ethanol. Solubility of clothianidin is also low in ethanol but this was resolved by allowing the mixtures of clothianidin and ethanol to sit for up to 3 days and confirming the absence of crystallization before coating bottles. For both insecticides, the slow acting effects meant that mosquitoes exposed in the bioassays had to be held for up to 3 days. This presents challenges as mosquitoes exposed in control tubes are more likely to die with increasing holding periods, which may invalidate the bioassays. Careful rearing procedures are required to ensure high quality bioassays with these slower acting insecticides.

## Conclusion

In summary, the estimated diagnostic doses for chlorfenapyr and clothianidin in CDC bottle bioassays as 50 ml/bottle and 150 ml/bottle respectively. Subsequently, the study demonstrated full susceptibility to these insecticides in wild populations of *An. gambiae*, *An. arabiensis*, and *An. funestus* that are resistant to pyrethroids. These results indicate that chlorfenapyr and clothianidin may be effective against the three main malaria vectors in western Kenya and they serve as a baseline for monitoring resistance should the Kenya National Malaria Control Programme implement LLINs or IRS using these insecticides.

## Additional file

**Additional file 1. Table S1.** Detailed results of the CDC bottle assay for different concentrations during determination of diagnostic concentrations for chlorfenapyr insecticide. **Table S2.** Detailed results of the CDC bottle assay performed for different concentrations during determination of diagnostic concentrations for clothianidin insecticides. **Table S3.** Control results for both chlorfenapyr and clothianidin insecticides respectively.

## Data Availability

The dataset supporting the conclusion is available from the corresponding author upon request.

## Abbreviations

CDC: Centre for Disease Control and Prevention
CGHR: Centre Global Health Research
GPIRM: Global Plan for Insecticide Resistance Management
LD: lethal dose
KEMRI: Kenya Medical Research Institute
WHO: World Health Organization
WHOPES: World Health Organization Pest Evaluation Scheme
