# Supplementary material for: Diagnostic dose determination and efficacy of chlorfenapyr and clothianidin insecticides against Anopheles malaria vector populations of western Kenya
Source: Malar J. 2019 Jul 17;18:243. doi: 10.1186/s12936-019-2858-z (PMC6637467; doi:10.1186/s12936-019-2858-z)
Supplement: Supplementary file 1 — Additional file 1. Table S1. Detailed results of the CDC bottle assay for different concentrations during determination of diagnostic concentrations for chlorfenapyr insecticide. Table S2. Detailed results of the CDC bottle assay performed for different concentrations during determination of diagnostic concentrations for clothianidin insecticides. Table S3. Control results for both chlorfenapyr and clothianidin insecticides respectively. [file 12936_2019_2858_MOESM1_ESM.docx]

Additional file 1:

**Table 1: Detailed results of the CDC bottle assay for different concentrations during determination of diagnostic concentrations for chlorfenapyr insecticide.**

| **Strain** | **Insecticide** | **Dose (µg/ml)** | **Number tested** | **Knockdown time**  **(0-60 minutes)** | | | | | | | **Mortality** | | |
| --- | --- | --- | --- | --- | --- | --- | --- | --- | --- | --- | --- | --- | --- |
|  |  |  |  | **0** | **10** | **20** | **30** | **40** | **50** | **60** | **24 hrs** | **48 hrs** | **72 hrs** |
| Kisumu | Chlorfenapyr | 10 | 400 | 0 | 0 | 0 | 0 | 0 | 0 | 0 | 75 | 93 | 101 |
| Kisumu | Chlorfenapyr | 20 | 400 | 0 | 0 | 0 | 0 | 0 | 0 | 0 | 128 | 163 | 170 |
| Kisumu | Chlorfenapyr | 30 | 400 | 0 | 0 | 0 | 0 | 0 | 0 | 0 | 329 | 358 | 364 |
| Kisumu | Chlorfenapyr | 40 | 400 | 0 | 0 | 0 | 0 | 0 | 0 | 0 | 367 | 381 | 387 |
| Kisumu | Chlorfenapyr | 50 | 400 | 0 | 0 | 0 | 0 | 0 | 0 | 0 | 391 | 400 | 400 |
| Kisumu | Chlorfenapyr | 60 | 400 | 0 | 0 | 0 | 0 | 0 | 0 | 0 | 394 | 400 | 400 |
| Kisumu | Chlorfenapyr | 70 | 400 | 0 | 0 | 0 | 0 | 0 | 0 | 0 | 397 | 400 | 400 |
| Kisumu | Chlorfenapyr | 80 | 400 | 0 | 0 | 0 | 0 | 0 | 0 | 0 | 397 | 400 | 400 |
| Kisumu | Chlorfenapyr | 90 | 400 | 0 | 0 | 0 | 0 | 0 | 0 | 0 | 393 | 400 | 400 |
| Kisumu | Chlorfenapyr | 100 | 400 | 0 | 0 | 0 | 0 | 0 | 0 | 0 | 387 | 398 | 400 |
| **Table 2: Detailed results of the CDC bottle assay performed for different concentrations during determination of diagnostic concentrations for clothianidin insecticides.** | | | | | | | | | | | | | |
| **Strain** | **Insecticide** | **Dose (µg/ml)** | **Number tested** | **Knockdown time**  **(0-60 minutes)** | | | | | | | **Mortality** | | |
|  |  |  |  | **0** | **10** | **20** | **30** | **40** | **50** | **60** | **24 hrs** | **48 hrs** | **72 hrs** |
| Kisumu | Clothianidin | 50 | 400 | 0 | 0 | 0 | 0 | 12 | 20 | 43 | 311 | 346 | 359 |
| Kisumu | Clothianidin | 100 | 400 | 0 | 0 | 0 | 0 | 12 | 20 | 46 | 320 | 346 | 376 |
| Kisumu | Clothianidin | 150 | 400 | 0 | 0 | 0 | 0 | 36 | 100 | 179 | 386 | 397 | 400 |
| Kisumu | Clothianidin | 200 | 400 | 0 | 0 | 0 | 11 | 58 | 181 | 243 | 391 | 397 | 400 |
| Kisumu | Clothianidin | 250 | 400 | 0 | 0 | 0 | 26 | 73 | 138 | 209 | 398 | 400 | 400 |
| **Table 3: Control results for both chlorfenapyr and clothianidin insecticides respectively.** | | | | | | | | | | | | | |
| **Kisumu** | **treatment** | **Dose(ml)** | **Number tested** | **Knockdown time**  **(0 – 60 minutes)** | | | | | | | **Mortality** | | |
|  |  |  |  | **0** | **10** | **20** | **30** | **40** | **50** | **60** | **24 hrs** | **48 hrs** | **72 hrs** |
| Kisumu | Absolute ethanol | 1ml | 200 | 0 | 0 | 0 | 0 | 0 | 0 | 0 | 10 | 12 | 12 |
| Kisumu | Absolute ethanol | 1ml | 200 | 0 | 0 | 0 | 0 | 0 | 0 | 0 | 12 | 13 | 15 |
